# *LOX* Gene Transcript Accumulation in Olive (*Olea europaea* L.) Fruits at Different Stages of Maturation: Relationship between Volatile Compounds, Environmental Factors, and Technological Treatments for Oil Extraction

**DOI:** 10.1100/2012/532179

**Published:** 2012-05-01

**Authors:** Innocenzo Muzzalupo, Barbara Macchione, Cristina Bucci, Francesca Stefanizzi, Enzo Perri, Adriana Chiappetta, Antonio Tagarelli, Giovanni Sindona

**Affiliations:** ^1^Centro di Ricerca per l'Olivicoltura e l'Industria Olearia, CRA, C/da Li Rocchi, 87036 Rende, Italy; ^2^Dipartimento di Chimica, Università della Calabria, 87036 Rende, Italy; ^3^Dipartimento di Ecologia, Università della Calabria, 87036 Rende, Italy

## Abstract

The quality of olive oil is influenced by genetic and environmental factors and by the maturation state of drupes, but it is equally affected by technological treatments of the process. This work investigates the possible correlation between olive *LOX* gene transcript accumulation, evaluated in fruits collected at different stages of maturation, and chemical biomarkers of its activity. During olive fruit ripening, the same genotype harvested from two different farms shows a positive linear trend between *LOX* relative transcript accumulation and the content of volatile compounds present in the olive oil aroma. Interestingly, a negative linear trend was observed between *LOX* relative transcript accumulation and the content of volatile compounds present in the olive pastes obtained from olive fruits with and without malaxation. The changes in the olive *LOX* transcript accumulation reveal its environmental regulation and suggest differential physiological functions for the LOXs.

## 1. Introduction

Lipoxygenases (LOXs) are widely distributed in the all plant kingdom and they exert diverse functions. LOXs are monomeric, nonheme iron-containing dioxygenases commonly found in animals, plants, and fungi [1]. Generally they are encoded by multigene families in complex eukaryotes [2]. In the last year, because of the rapid progress in structural and functional genomics research, *LOX* genes are being identified and studied in an increasing number of animal and plant species. They have a role as storage proteins in seeds [3] and regulatory functions in some aspects of plant development such as potato tuber growth [4]. LOXs are also involved in plant-fungus interaction in peanut [5]. Interestingly, the oxylipins, the end-products of fatty acid oxidation, mediated by LOXs action, are specifically involved in signalling and plant defence responses.

**Figure 1:**
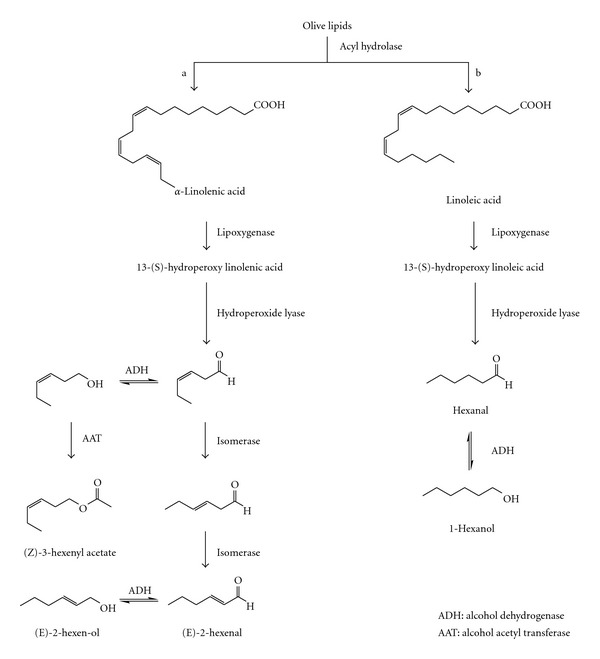
Scheme of the lipoxygenase metabolic pathway.

LOX proteins constitute an important class of lipid-hydrolyzing enzymes that catalyze the oxygenation of polyunsaturated fatty acids such as linolenic (LnA) and linoleic acids (LA) (Figure 1) [6, 7].

LOXs have also a role in the production of volatile molecules that can positively or negatively influence the flavour and aroma in many plant products such as in apple [8, 9], strawberry [10], and pear fruit [11].

In plants, LOXs are divided into two types, 9-LOXs and 13-LOXs, which catalyze oxygenation at the carbon atoms 9 and 13 of the hydrocarbon backbone of LA, respectively. However, this classification scheme has encountered difficulty because more and more animal and plant LOXs are found to yield a mixture of products upon catalysis [12–14]. Traditionally, animal and plant LOXs are named and classified according to the specificity of their expression pattern, subcellular location, and action on the substrate [1, 15, 16]. LOXs are considered of a great interest in food science because free radical processes, resulting from their activity, can exert deleterious effects on nutritionally important compounds such as essential polyunsaturated fatty acids [17].

In olive (*Olea europaea *L.) fruits, the LOX pathway is responsible for the production of desirable organoleptic properties that differentiate virgin olive oil from other vegetable oils. Hexanal (E)-2-hexenal, (E)-2-hexen-1-ol, 1-hexanol, and (*Z*)-3-hexen-1-yl acetate are five biomarkers produced as a consequence of lipid degradation following tissue disruption, and they are among the most important volatile compounds in olive oil aroma [18–20]. Dhifi et al. [21] has reported that the qualitative and quantitative composition of the olive oil aroma is tightly dependent on the enzymatic store, involved in the LOX pathway, which is linked to fruit ripening. Olive fruit growth and development takes place in ~5 months after flowering, depending on the variety and climatic condition, and it includes different phases such as cell division, cell expansion, and storage of metabolites [16]. The quality of olive oil is influenced by genetic and environmental factors and by the maturation state of drupes, but it is equally affected by technological treatments such as malaxation [22, 23]. Malaxation for 20 to 40 minutes allows small oil droplets to combine into bigger ones which can be removed by centrifugation. Centrifugation is a step absolutely necessary for effective extraction of the oil [22]. Longer mixing increases the oil yield and allows the formation of minor components that enhance its flavour, but it produces more oxidation products which make the acidity and peroxide values of oil higher, shortening its shelf life [23]. It has been stated that in olive fruit an enzymatic system is present, which is genetically determined, including acylhydrolase (AH), LOX, fatty acid hydroperoxide lyase (FAHL), alcohol dehydrogenase (ADH), and alcohol acyltransferase (AAT). It becomes quickly active upon cell disruption and it is involved in the formation of green sensory notes, covering the range between sweet-fruity-green to bitter-powerful-green [16]. Thus, the process of obtaining olive oil can be considered a good example of a system that produces secondary green volatiles.

In this work, the possible correlation between olive *LOX* gene transcript accumulation and chemical biomarkers of its activity was investigated. The genotype analyzed was the “Coratina”, cultivar originating from Apulia region, where it is present on over 70,000 hectares. Most “Coratina” plants are mainly located in the Southern Italian region, but they are widely distributed throughout the national territory [24]. The virgin olive oils investigated in this work are all obtained from (i) a single olive genotype; (ii) harvested in two different farms located in the Calabria region; (iii) collected in three ripening stages. Changes in composition of the volatile fractions of virgin olive oils produced from “Coratina” olive pastes with and without malaxation were also evaluated.

## 2. Materials and Methods

### 2.1. Olive Sampling and Origin Areas

**Figure 2:**
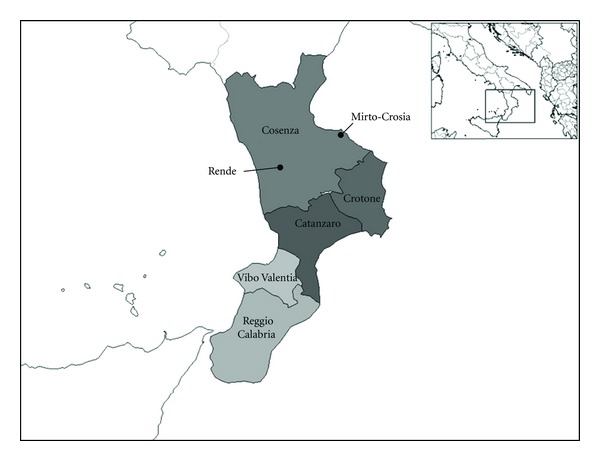
Map of areas where samples were collected. The samples come from two different growing places of the Calabria region (Italy).

The selection of plants of “Coratina” cv, belonged to the olive germplasm collection of the CRA-OLI (Centro di Ricerca per l'Olivicoltura e l'Industria Olearia), was performed in the town of Mirto-Crosia (Jonian coasts, Calabria, Italy) and in the town of Rende (Internal zone, Calabria, Italy) (Figure 2). In each farm the olive fruits were collected from five olive trees during three ripening stages: at 170 days after flowering (DAF, *green mature*), at 200 DAF (*black with <50% purple flesh*), and at 230 DAF (*black with >50% purple flesh*). The collected olive samples were used for all subsequent experiments. All “Coratina” plants showed the same genotype when DNA fingerprinting by microsatellite markers was performed and therefore were indistinguishable from each other [25].

### 2.2. RNA Isolation and Reverse Transcriptase

Total RNA was isolated from olive tissues at different developmental stages and processed separately. Frozen tissues, obtained using liquid nitrogen (100 mg), were processed with the RNeasy Plant Mini kit (Qiagen, Hilden, Germany) according to the manufacturer's instructions. In the elution step, RNA was resuspended in a volume of 50 *μ*L of RNase free water and incubated at 37°C for 30 min with 2 unit of DNase I in a final volume of 100 *μ*L. DNAse I was inactivated at 70°C for 15 min.

RNA was precipitated and finally resuspended in 40 *μ*L of RNase-free water. Quality and quantity of total isolated RNA were controlled with a NanoDrop Spectrophotometer ND-1000. The total RNA (35 *μ*g) from each sample was used, with the high-capacity cDNA reverse transcription (Applied Biosystems, Applera, Monza, Italia), according to the manufacturer's instructions (Applied Biosystem).

### 2.3. Quantitative Real-Time PCR (qRT-PCR)

qRT-PCR was performed on a Applied Biosystems 7500 Real Time PCR Systems (Applied Biosystem). Single colour thermocycler with *Power *SYBR Green PCR Master Mix 2X was used (Applied Biosystem).

### 2.4. Primer Design

The oligonucleotide primer sets used for qRT-PCR analysis were designed using Primer Express version 3.0 (Applied Biosystem) according to the strategies set up by Muzzalupo et al. [26]. Experimentally optimal primers were identified based upon their ability to meet several standards: (a) robust, successful amplification over a range of annealing temperatures, (b) specificity generation of a single significant peak in the melting curve, and (c) consistent, highly reproducible *Ct* values within the reactions of a triplicate. The primers used for *LOX* gene (GenBank EU678670) were forward (Fw) real time 5′-TCCCATTGCCTCAGGTTATCA-3′ and backward (Bw) real time 5′-TCTCTCGCGAATTCTTCATCTG-3′. The length of all PCR products ranged from 150 to 200 bp. The average amplification efficiency of each primer pair was determined, and primers performing poorly were replaced. The average efficiency of all the primer pairs discussed in this study ranged between 0.95 and 1.0. After checking independent trials of several housekeeping genes, 18S rRNA produced the most reproducible results across various cDNAs, and was used as a normalization control. The primer sequence of 18S rRNA was Fw 18S 5′-AAACGGCTACCACATCCAAG-3′ and Bw 18S 5′-CCTCCAATGGATCCTCGTTA-3′.

### 2.5. Amplification Conditions

Amplification reactions were prepared in a final volume of 25 *μ*L by adding 12.5 *μ*L of *iTaq* SYBR-Green Super Mix with ROX (Bio-Rad Laboratories S.r.l., Rome, Italy) containing the (*iTaq* DNA polymerase 50 units mL^−1^, 6 mm Mg^2+^, 1 *μ*M ROX internal Reference DYE Stabilisers, 0.4 mM of dATP-dCTP-dGTP, and 0.8 mM dUTP) 0.4 *μ*M of primers, and 2 *μ*L (25 ng) of cDNA. All reactions were run in triplicate in 48-well reaction plates, and negative controls were set. The cycling parameters were as follows: one cycle at 95°C for 3 min to activate the *Taq *enzyme, followed by 40 cycles of denaturation at 95°C for 10 s and annealing-extension at 58°C for 30 s. After reaction, in order to confirm the existence of a unique PCR product, the *melting curve *[27] was evaluated by an increase of 0.5°C every 10 s, from 60°C to 95°C. We obtained a unique “melting peak” in every reaction, and the PCR products were verified by 1% agarose gel electrophoresis.

### 2.6. qRT-PCR Data Analysis

The results of qRT-PCR were analysed using Opticon Monitor: qRT-PCR Detection System (Bio-Rad), a program that facilitates the analysis of the kinetics of each performed reaction. Cycle threshold (*Ct*) values were obtained with the Genex software (Bio-Rad), and data were analysed with the 2^DDCT^ method [28]. The means of *LOX *transcript accumulation was calculated from three biological repeats, obtained from three independent experiments.

### 2.7. Olive Oil Extraction

For each sample 5 kg of olives were picked from five trees grown in both farm considered (Rende e Mirto-Crosia), which were homogeneous for cultivar and health, and then milled in a laboratory scale hammer mill (Oliomio, Toscana Enologica Mori, Tavernelle Val di Pesa, Italy). After 30 min of malaxation at room temperature, the oil was separated by centrifugation in the same operative conditions.

Olive pastes without malaxation (*OP1*), olive pastes after 30 min of malaxation (*OP2*), and olive oil obtained after centrifugation were stored at −18°C until volatile fraction analysis were performed.

### 2.8. Analysis of Volatile Compounds

The preparation of samples and the most suitable solid-phase microextraction (SPME) conditions for quantitative assay of the five selected compounds, hexanal, (*E*)-2-hexenal, (*E*)-2-hexen-1-ol, 1-hexanol, and (*Z*)-3-hexen-1-yl acetate were described by a previous study [7]. Briefly, 2 g of olive paste (*OP1* and *OP2*) or olive oil samples were placed in a septum-closed vial, and the extraction was performed in the headspace volume (~8 mL) at 40°C for 20 min by a SPME divinylbenzene/carboxen/polydimethylsiloxane (DVB/CAR/PDMS) 65 *μ*m fibre (Supelco Co., Bellefonte, PA). The adsorbed analytes were thermally desorbed by introducing the fibre into the injector set at 250°C for 3 min.

Volatile fraction analysis was performed using a Varian (Walnut Creek, CA) Saturn 2000 GC-MS ion trap (ITD) system in positive CI mode, with iso-butane as the reagent gas, coupled to a Varian 3400 gas chromatograph equipped with a Varian 8200 autoinjector. The ion trap temperature was set at 210°C with an ionization time of 2 ms, a reaction time at 50 ms, and a scan rate at 1000 ms. The transfer line temperature was set at 230°C. The column was a 30 m Varian Factor Four 5-ms (0.25 mm i.d., 0.25 *μ*m film thickness). The gas chromatography (GC) oven temperature was initially held at 40°C for 3 min, then increased at 1°C min^−1^ to 70°C, increased again at 20°C min^−1^ to 250°C, and held for 8 min. The carrier gas was helium at 1 mL min^−1^. Analyses were performed in split less mode. For SPME analyses, a narrow-bore Supelco 0.8 mm i.d. GC inlet liner was used. The quantitative assay was performed in chemical ionization mode, using isobutane as the reagent gas and ethyl isobutanoate as internal standard at the concentration ranges 0.2–2 and 5–100 mg kg^−1^, using 1 and 40 mg kg^−1^ of IS, respectively. The mean values of volatile compounds were calculated from three biological repeats, obtained from three independent experiments.

### 2.9. Other Parameters

The preparation of methyl esters of the fatty acids was carried out according to the EC Official Gazette (EEC no. 2568/91). A gas chromatograph (GC System Agilent 6890 N Network Technologies, Rome, Italy) was employed with a capillary column Supelco SP-2380 (0.35 mm i.d. 0.25 m film thickness, silica phase) and a flame ionization detector (FID). The gas chromatographic conditions were as follows: oven temperature programmed from 160 to 174°C at 2°C min^−1^ and from 174 to 190°C in 15 min at 4°C min^−1^. FID temperature was set at 250°C and H_2_ carrier gas pressure at 18 psi.

The phenolic compounds were determined according to Folin-Ciocalteu method [29] using caffeic acid as standard. The oil content was determined by Soxhlet extraction using hexane for 6 h. After evaporation of solvent, the oil content was determined gravimetrically.

### 2.10. Statistical Analysis

All parameters analysed were carried out in triplicate. The results are reported as the mean values obtained from three repetitions and their standard deviation. Significant differences among samples were determined by analysis of variance which applied a Duncan test with a 95% significant level (*P* < 0.05), using the IBM SPSS statistics professional edition software 11.0 for Windows.

## 3. Results and Discussion

LOXs activity increases during ripening processes such as loss of firmness in kiwifruit [30] and peach fruit [31] thus contributing to membrane deterioration in plant tissue, through peroxidation of polyunsaturated fatty acids. In this context, LOXs are considered of a great interest in food science because the free radicals produced via the LOX pathway during cell degradation can exert deleterious effects on nutritionally important compounds, such as the essential polyunsaturated fatty acids [32].

As a consequence of the cell degradative processes that occurred in fruit ripening related to LOXs activity, there is the production of C6 alcohols and aldehydes, volatile molecules that positively or negatively influence the flavour and aroma in many plant products. These compounds are principally formed from 13-LOXs group of enzymes which produce 13-fatty acid hydroperoxides that subsequently are converted into hexanals and their alcohols (Figure 1).

### 3.1. LOX Transcript Accumulation

The transcript accumulation of a type-1 *LOX* gene during olive ripening was monitored in order to clarify the role of *LOX* genes in olives and their contribution to the development of the olive oil aroma. In this work, the *LOX* mRNA accumulation was considered as equilibrium between synthesis and degradation of the *LOX* gene transcripts. *LOX *transcript accumulation was estimated through qRT-PCR analysis in fruits harvested at three ripening stages (170 DAF, 200 DAF, and 230 DAF).

**Figure 3:**
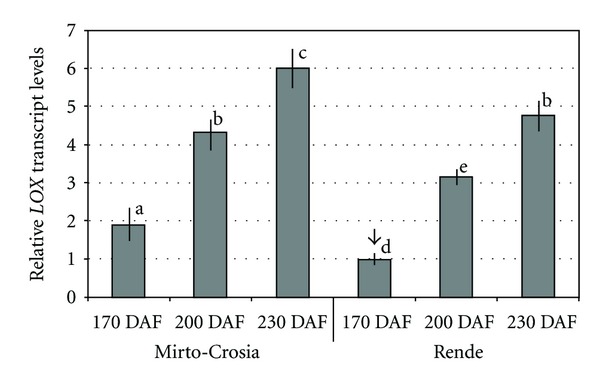
Transcript accumulation of *LOX* gene in “Coratina” cv fruits harvested at three stages of ripening (170 days after flowering DAF = green mature; 200 DAF = black with <50% purple flesh; 230 DAF = black with >50% purple flesh) and in two different cultivation areas (Rende and Mirto-Crosia). To “Coratina” samples collected on the 170 DAF stage, (↓) was assigned the value of 1.0 and it was used as calibrators. Data are expressed as mean values ± standard deviation of three independent experiments. Duncan test has been used to assess significance (*P* = 0.05). Values followed by different letters in the same line are significantly different.

It was found that *LOX* transcripts were differentially expressed in samples collected in both farms and increased during fruits ripening. Most of the obtained values were statistically different (Figure 3). In particular, the lesser values were detected in 170 DAF fruits collected from the farm located in Rende and then used as calibrators assigning the value of 1.0 (Figure 3). Furthermore, *LOX *transcripts were 5-fold more abundant in fruit collected at 230 DAF in the Mirto-Crosia area than the calibrator. The same trend, in the relative level of gene transcript accumulation, was observed in the drupes harvested in Rende, but it shows lower values with respect to that observed in Mirto-Crosia. It is worth noting that the farm located in Mirto-Crosia, being located near the sea, has a milder climate, with temperatures always warmer than the Rende farm (data provided by website http://www.ilmeteo.it/meteo/). This aspect could be a possible cause of the increase in plant metabolism and *LOX* transcript accumulation observed in olive fruits.

Also, low levels of *LOX* gene transcript accumulation were reported at the early stages of the olive ripening and increased levels in the black stage. These results, according to Padilla et al. [16], are not surprising since LOXs are linked with plant senescence and fruit ripening. In our case, during olive fruit ripening, the same genotype harvested from two different farms shows a positive linear trend between *LOX* relative transcript accumulation and the content of volatile compounds present in the olive oil aroma. Interestingly, a negative linear trend was observed between *LOX* relative transcript accumulation and the content of volatile compounds present in the olive pastes obtained from olive fruits, with and without malaxation (Figure 3 and Table 1).

### 3.2. Volatile Compounds Analysis

According to literature [19, 21, 32–34], the following five secondary metabolites were selected as markers of the lipoxygenase oxidation pathway: hexanal, (E)-2-hexenal, 1-hexanol, (E)-2-hexen-ol, and (*Z*)-3-hexenyl acetate. The quantitative data show substantial differences between samples produced in the two considered Calabria areas. Moreover, a significant variation has been observed in samples with different fruit ripening stages (Tables 1 and 2).

As reported in Table 1, (E)-2-hexenal content is by far the highest in all samples in accordance with previous research results [19]. A significant trend observed in the data of this study is represented by the highest level of volatile compounds in 170 DAF fruits in both considered areas and a steady decrease during the progression of fruit ripening. Besides, the *LOX* gene showed an increased accumulation of transcripts in the olive fruit during ripening (Figure 3), which coincides with a decrease in the synthesis of volatile compounds as detected in olive paste and olive oil aroma. This result indicates that the *LOX* gene was expressed at late developmental stages of the olive ripening, suggesting that it is probablly linked with the senescence process. Although its contribution to the biosynthesis of the olive oil aroma cannot be ruled out, it exhibits LOX activity in a 2 : 1 ratio with LA as substrate, as suggested also by Palmieri-Thiers et al. [7].

The concentration levels of (E)-2-hexenal are greater in olive oil than in the equivalent olive paste obtained with or without malaxation, regardless of the zone of olive cultivation. In fact, the volatile compounds formed during crushing and malaxation steps are accumulated into oil generating the characteristic aroma. Besides, using these characteristic metabolic profiles (hexanal, E-2-hexenal, and other aldehydes) in combination with genetic and spectroscopic analyses allows us to set up a strategy for a cultivar and/or geographical origin discrimination of the extra virgin olive oils [35].

The olive oil samples collected in each area of olive plants cultivation considered showed the maximum levels of (E)-2-hexenal in 200 DAF fruits. An elevated concentration of (E)-2-hexenal was found in all samples obtained from Rende areas, with respect to samples obtained from Mirto-Crosia area (Table 2). These results indicated that LnA was clearly the preferred substrate of LOX enzyme for both environments.

All olive paste samples of Mirto-Crosia area showed a higher content of hexanal+1-hexanol than paste samples obtained from Rende area (Table 1), and this behaviour was even more pronounced for the olive oil sample (Table 2). The (E)-2-hexen-1-ol was present only in olive oils at 170 and 230 DAF fruits obtained from Mirto-Crosia area (Table 2). This aspect might be due to an over-activity of the dehydrogenase (ADH) enzymes in these samples. Finally in all samples, the (*Z*)-3-hexen-1-yl acetate was not detected.

### 3.3. Effects of Malaxation on the Volatile Composition?

Tables 1 and 2 show the data obtained monitoring the selected volatile compounds in samples of olive pastes kneaded with and without malaxation (*t* = 0 and *t* = 30 min) (Table 1) and in olive oils obtained after 30 min of malaxation (Table 2). It is important to point out that the amount of the volatile compounds changes with the prolonging of malaxation step, and with the olive ripening stage. In particular, the most important change was found for (E)-2-hexenal. For this compound we noticed that prolongation in malaxation time (from 0 to 30 min) determined a significant decrease in concentration of this aldehyde in paste samples, collected at 170 DAF. An opposite trend was observed in the olive paste at 200 DAF in which it was found that (E)-2-hexenal content significantly increased after 30 min of malaxation time.

In 230 DAF fruits, no significant variations in (E)-2-hexenal concentration for both areas of cultivation were observed. This trend may be due to the different degree of inactivation of enzymes. In fact, LOX enzyme can be influenced by the different phenolic levels monitored in the fruit at the various stages of ripening. The inactivating role of phenolic compounds on enzyme activity is well established [36]. In the olive oils obtained from 170 DAF fruits in each area, phenolic compounds were significantly greater in 170 DAF with respect to 200 and 230 DAF fruits (Table 2).

### 3.4. Other Parameters

Phenols and lipids contribute to determine the nutritional qualities of vegetable oils, but the fatty acid composition is also very important. In fact, monounsaturated fatty acids, such as oleic acid, exert beneficial effects in cardiovascular disease prevention [37]. Consequently, the increase of oleic acid has become one of the major goals to improve vegetable oil quality. However, it is the presence of minor components, in particular phenols, contributing to oil's high oxidative stability, to colour and flavour, that makes olive oil unique among other oils.

The pattern of the parameters investigated (Table 2) showed that the ripeness stage of “Coratina” olives that yields the best quality of oil corresponds to the 200 DAF fruits. In fact, the oils produced by olives harvested at this time frame (200 DAF fruits) are characterized by high nutritional properties (i.e., oleic acid and total phenol content).

## 4. Conclusions

The results show that the *LOX* transcript accumulation increased during olive fruit ripening on the “Coratina” genotype cultivated in two different farms. This trend suggests that it is connected with ripening and senescence processes. In Mirto-Crosia farm, the *LOX *transcripts in olive fruits were significantly more abundant than in the Rende farm. The difference of *LOX* transcript accumulation between the Mirto-Crosia and Rende farms may be due to environmental differences (i.e., temperature, soil fertility, humidity, etc.). Olive paste and olive oil samples, obtained by fruits collected in both farms, showed different volatile contents. The volatile molecules are produced via the LOX pathway during the mechanical oil extraction process and represent the main classes of compounds responsible for “fruity”, “cut-grass”, and “floral” flavours. Although the response of the volatile compound concentrations depended mainly on the growing season, there were some consistent trends which could be attributed to environmental differences. In particular, the concentrations of hexanal, (E)-2-hexenal, and 1-hexanol were significantly affected by humidity, similar to results reported by Servili et al. [38].

The data in this study show that the production of volatile molecules strictly linked to environmental parameters. In fact, (E)-2-hexenal content is, in all samples analyzed, by far the highest in accordance with the LOX preference for LnA fatty acid. The presence of hexanal and 1-hexanol in olive paste and in olive oils samples obtained by fruits collected in Mirto-Crosia farm suggests also a good activity of the dehydrogenase (ADH) enzymes. In fact, the oil aroma is determined by all enzyme activities involved in the LOX pathway.

The changes in olive *LOX* transcript accumulation reveal, for the first time, its environmental regulation and suggest differential physiological functions for the LOXs. The results herein reported suggest that a multidisciplinary approach could be used to set up a method for geographic origin certification, based on the construction of a suitable database [39].

This information suggests that specific *LOX* gene in olive fruit may be involved in fruit ripening, with consequences for flavour or aroma development in the virgin olive oils.

## Figures and Tables

**Table 1:** Content of five volatile biomarkers: (2-(E)-hexenal, hexanal, 1-hexanol, (E)-2-hexen-1-ol, and (*Z*)-3-hexen-1-yl acetate) analyzed in the olive pastes obtained from “Coratina” cv. Samples were performed with and without malaxation (*OP1*, *t* = 0 min; *OP2*, *t* = 30 min). The drupes were collected at three developmental stage of ripening (170 days after flowering DAF = green mature; 200 DAF = black with <50% purple flesh; 230 DAF = black with >50% purple flesh) and in two different cultivation areas (Rende and Mirto-Crosia). Data on volatile biomarkers contents from olive paste are expressed in mg/kg. Data are expressed as mean values ± standard deviation of three independent experiments. Duncan test has been used to assess significance (*P* = 0.05). Values followed by different letters in the same line are significantly different. n.d.: not detected.

Volatile compounds (mg kg^−1^)	Mirto-Crosia					
	*170 DAF*		*200 DAF*		*230 DAF*	
	*OP1*	*OP2*	*OP1*	*OP2*	*OP1*	*OP2*
hexanal	1.89 ± 0.39^a^	2.50 ± 0.72^a^	2.32 ± 0.60^a^	2.04 ± 0.61^a^	0.93 ± 0.22^b^	1.42 ± 0.31^b^
(E)-2-hexenal	17.39 ± 1.55^a^	12.68 ± 1.17^b^	10.42 ± 0.82^c^	14.26 ± 1.04^d^	8.61 ± 0.76^e^	7.17 ± 0.81^e^
(E)-2-hexen-1-ol	n.d.	n.d.	n.d.	n.d.	n.d.	n.d.
1-hexanol	0.92 ± 0.09^a^	0.93 ± 0.08^a^	0.99 ± 0.10^a^	1.03 ± 0.10^a^	0.45 ± 0.05^b^	0.53 ± 0.05^b^
(*Z*)-3-hexen-1-yl acetate	n.d.	n.d.	n.d.	n.d.	n.d.	n.d.

	Rende					
	*170 DAF*		*200 DAF*		*230 DAF*	
	*OP1*	*OP2*	*OP1*	*OP2*	*OP1*	*OP2*

hexanal	0.25 ± 0.08^a^	0.21 ± 0.07^a^	0.41 ± 0.07^a^	0.28 ± 0.07^a^	0.66 ± 0.18^b^	0.61 ± 0.17^b^
(E)-2-hexenal	19.91 ± 1.18^a^	15.01 ± 1.02^b^	14.53 ± 0.92^b^	16.95 ± 0.98^b^	11.67 ± 1.17^c^	10.48 ± 1.04^c^
(E)-2-hexen-1-ol	n.d.	n.d.	n.d.	n.d.	n.d.	n.d.
1-hexanol	n.d.	0.91 ± 0.09^a^	0.91 ± 0.09^a^	0.92 ± 0.09^a^	0.98 ± 0.10^a^	0.98 ± 0.10^a^
(*Z*)-3-hexen-1-yl acetate	n.d.	n.d.	n.d.	n.d.	n.d.	n.d.

**Table 2:** Virgin olive oil quality indices from “Coratina” cv after malaxation (*t* = 30 min). The yield in oil (in % on dry matter), the content of volatile biomarkers (in mg kg^−1^ oil), the main fatty acids (in %), and the total phenols (in mg kg^−1^ in caffeic acid) are reported. The drupes were collected at three developmental stage of ripening (170 days after flowering DAF = green mature; 200 DAF = black with <50% purple flesh; 230 DAF = black with >50% purple flesh) and in two different cultivation areas (Rende and Mirto-Crosia). Data are expressed as mean values ± standard deviation of three independent experiments. Duncan test has been used to assess significance (*P* = 0.05). Values followed by different letters in the same line are significantly different. n.d.: not detected.

		Mirto-Crosia			Rende		
		*170 DAF*	*200 DAF*	*230 DAF*	*170 DAF*	*200 DAF*	*230 DAF*
Yield in oil (% dry matter)		42.7 ± 1.9^a^	47.8 ± 2.3^b^	50.3 ± 2.8^b^	41.2 ± 2.2^a^	46.2 ± 2.8^b^	50.0 ± 2.2^b^

	hexanal	1.66 ± 0.52^a^	2.56 ± 0.75^ab^	1.52 ± 0.49^a^	0.51 ± 0.11^c^	0.46 ± 0.09^c^	0.79 ± 0.19^c^
	(E)-2-hexenal	20.2 ± 1.6^a^	25.8 ± 2.6^ab^	18.5 ± 1.8^ac^	38.8 ± 2.2^d^	45.2 ± 3.0^e^	24.4 ± 2.4^ab^
Volatile compounds (mg kg^−1^)	(E)-2-hexen-1-ol	1.58 ± 0.58^a^	n.d.	1.45 ± 0.52^a^	n.d.	n.d.	n.d.
	1-hexanol	0.96 ± 0.09^a^	0.93 ± 0.09^a^	0.88 ± 0.08^a^	n.d.	0.92 ± 0.09^a^	0.91 ± 0.09^a^
	(*Z*)-3-hexen-1-yl acetate	n.d.	n.d.	n.d.	n.d.	n.d.	n.d.

	Palmitic ac. (C16 : 0)	10.89 ± 0.89^a^	10.52 ± 0.99^a^	10.04 ± 0.78^a^	12.3 ± 1.0^ab^	10.94 ± 0.99^a^	10.73 ± 0.96^a^
	Palmitoleic ac. (C16 : 1)	0.75 ± 0.07^a^	0.70 ± 0.05^a^	0.68 ± 0.05^a^	0.72 ± 0.06^a^	0.65 ± 0.05^a^	0.62 ± 0.05^a^
Fatty acids (%)	Stearic ac. (C18 : 0)	2.01 ± 0.12^a^	1.96 ± 0.11^a^	1.77 ± 0.11^a^	2.66 ± 0.12^a^	2.02 ± 0.12^a^	1.80 ± 0.11^a^
	Oleic ac. (C18 : 1)	78.5 ± 1.5^a^	79.1 ± 1.6^a^	79.6 ± 1.7^a^	76.67 ± 0.43^a^	78.6 ± 1.6^a^	78.8 ± 1.7^a^
	Linoleic ac. (C18 : 2)	6.05 ± 0.35^a^	6.36 ± 0.38^a^	6.68 ± 0.41^a^	6.38 ± 0.39^a^	6.60 ± 0.39^a^	6.97 ± 0.42^a^
	*α*-Linolenic ac. (C18 : 3)	0.92 ± 0.11^a^	0.81 ± 0.09^a^	0.72 ± 0.09^a^	0.94 ± 0.09^a^	0.84 ± 0.09^a^	0.75 ± 0.09^a^

Total phenols (mg kg^−1^ in caffeic acid)		450 ± 63^a^	315 ± 35^b^	157 ± 22^c^	510 ± 72^a^	327 ± 42^b^	182 ± 33^c^
